# Technology for Mental Health: Reflections on Scope and Future Directions in Institutes of Higher Education in India

**DOI:** 10.2196/78065

**Published:** 2025-11-21

**Authors:** Seema Mehrotra, TK Srikanth, Neha Dahiya, Pulkit Verma, Girish N Rao, Prachi Sanghvi, Ashoo Grover, Rajesh Sagar

**Affiliations:** 1 Department of Clinical Psychology, National Institute of Mental Health and Neurosciences Bengaluru, Karnataka India; 2 E-Health Research Centre, International Institute of Information Technology Bengaluru, Karnataka India; 3 Division of Delivery/ Implementation Research, Indian Council of Medical Research (ICMR) New Delhi India; 4 Informatics and Data Centre Division, Indian Council of Medical Research (ICMR) New Delhi India; 5 Department of Epidemiology Centre for Public Health National Institute of Mental Health and Neurosciences Bangaluru, Karnataka India; 6 Department of Clinical Psychology Rashtriya Raksha University Gandhinagar, Gujarat India; 7 Department of Psychiatry All India Institute of Medical Sciences New Delhi, New Delhi India

**Keywords:** digital mental health, higher education, India, Institutes of Higher Education, student mental health, technology for mental health

## Abstract

College years represent a pivotal phase as students transition into adulthood, a period marked by heightened vulnerability to mental health challenges. Beyond the high prevalence of common mental health issues, a large treatment gap (driven by both supply-side and demand-side factors) exacerbates the overall burden. Furthermore, students in higher education frequently experience psychological distress and subthreshold symptoms that impair well-being and daily functioning. Globally, technology-based mental health solutions have emerged as an important strategy to address unmet needs, with a growing evidence base across populations. Research has increasingly focused on examining digital mental health interventions for college students. Against this backdrop, we examine the challenges within India’s large higher education system—which serves approximately 43 million students—and the expanding role of technology in this sector. We explore the potential for leveraging technology-based solutions to enhance student mental health initiatives within higher education institutions, considering relevant policies and guidelines that provide an impetus to these efforts. We reflect upon challenges and opportunities for implementing digital mental health interventions in Indian higher education, and propose strategic actions at institutional and governmental levels. Key considerations include data governance, safety, transparency, positioning of digital initiatives relative to in-person care, safeguards for content quality, provision of interventions at varying intensities, and recommendations for policy, governmental support, and research to optimize the use of technology for student mental health in institutes of higher education in India.

## Mental Health of Students in Universities

College years mark a crucial phase in the transition to emerging adulthood as individuals advance in their individuation journey, expand social connections, and explore their identities. These years also introduce numerous demands, including heightened academic pressures, career planning, adaptation to new social environments, formation of peer networks, and navigation of close relationships. Most college students experience stress in one or more life domains, such as finances, health, intimacy, relationships with family, relationships on campus, and problems experienced by significant others [1].

Youth and young adulthood constitute a vulnerable period, as 75% of mental health conditions have an onset before the age of 24 years [2]. The World Health Organization (WHO) World Mental Health Surveys conducted in 21 countries with varied income levels indicated that 20.3% of college students had a 12-month prevalence of diagnosable mental health disorders [3]. Nearly one-third of first-year university students from 19 universities across 8 countries screened positive for at least 1 common mental disorder including anxiety, mood, or substance disorder as per the *DSM-IV* (*Diagnostic and Statistical Manual of Mental Disorders* [Fourth Edition]*)* [4]. Beyond diagnosable conditions, subthreshold symptoms of common mental health conditions, such as depression, are also highly prevalent (eg, affecting up to 29% of youth) and can impair functioning, social activity, and performance, as well as increase the risk of major depressive disorder and suicide attempts [5]. An international study among undergraduate students across 12 countries in Europe, Asia, the Western Pacific, and North and Latin America found that 48% of participants exhibited clinically significant depressive symptoms [6]. In addition to common mental health conditions, rising youth suicide rates have drawn considerable attention, underscoring the urgent need for action. According to the WHO, suicide is the fourth leading cause of death among persons aged 15-29 years [7]. In India, the 18- to 29-year age group has the highest suicide deaths among both young men and women [8,9]. Student suicides constituted 7.6% of total suicides in the country, similar to the proportion among unemployed persons [8]. Over the last 10 and 20 years, total suicides increased by an average of 2% annually, whereas student suicides increased by 4%—twice the rate of total suicides [10]. For every death by suicide in India, more than 200 people experience suicidality in some form (eg, suicidal thoughts) and more than 15 suicide attempts occur [11]. A large-scale study across 9 Indian states found that one-fourth of college students had heard someone express suicidal ideas, 12.4% experienced suicidal thoughts in the past 12 months, and 6.7% reported a suicide attempt at some point in their lives; in the same study, one-third reported moderate-to-severe depressive symptoms and one-quarter reported moderate-to-severe anxiety [12].

There is voluminous research on the effectiveness of interventions for various mental health problems. For example, an umbrella review encompassing 31 meta-analyses and 608 primary studies evaluated psychological interventions for preventing and treating common mental disorders in college students and noted the availability of effective universal, indicated, and treatment-based interventions for both depression and anxiety [13]. Despite the availability of effective interventions, a substantial treatment gap persists globally and in India. Findings from the National Mental Health Survey of India 2015-2016 indicated a treatment gap of about 80% for common mental disorders [14]. A United Nations Children’s Fund report (2021) highlighted that only 41% of youth aged 15-24 years surveyed in India felt the need to seek support when facing mental health challenges [15].

The World Mental Health Surveys further revealed that only 16.4% of students experiencing diagnosable levels of mental health problems received any treatment over a 12-month period [3]. In addition, a large international study of first-year students from 19 universities across 8 countries reported low treatment rates over the same timeframe: only 25.3% of students with mental health disorders and 29.5% of those experiencing suicidal thoughts and behaviors received care [16].

Not seeking help for common mental health issues in young people can lead to short- and long-term negative outcomes, including diminished educational and career prospects and an increased risk of developing comorbid conditions [17,18]. Multiple factors contribute to the treatment gap, including limited access to appropriate mental health care and insufficient or inequitably distributed resources (supply-side barriers), as well as demand-side barriers such as low perceived need, stigma, and adverse attitudes toward help-seeking [14,19]. Among students, additional barriers include stigma, fear of negative consequences for academic records, normalization of distress as a passing phase, preference for informal sources of help, low prioritization amid other commitments, skepticism about the utility of professional services, and inconvenient access [20,21].

These observations underscore the urgent need to address supply-side barriers by improving access to services as well as to tackle demand-side challenges by developing approaches that facilitate early identification and professional help-seeking. Past efforts have predominantly targeted access by reducing supply-side barriers, whereas demand-side obstacles have received comparatively less attention because of their complexity [22]. Given the multiple barriers to professional help-seeking documented among young adults, including students, there is a pressing need for innovative, culturally sensitive, acceptable, and scalable approaches to address unmet mental health needs [18,20,23].

## Role of Technology for Mental Health in General and in Young Adults

Integration of technology into mental health services has expanded accessibility, outreach, and intervention delivery worldwide [24]. With increasing internet penetration and advances in digital tools—particularly in low- and middle-income countries (LMICs) such as India, there is an immense potential to address the mental health needs of diverse populations. Technology can also partly mitigate barriers related to shortages in the mental health workforce and enhance the scalability of interventions.

Globally, there has been an exponential increase in internet penetration, including in resource-limited countries. According to a joint report from the Internet and Mobile Association of India and KANTAR, the number of internet users in India has surpassed 800 million [25]. India is expected to achieve universal internet access by 2028-2029 [26]. Increased technology use can expand the reach and scope of mental health services [27]. Young adults, in particular, show strong interest in technology-enabled tools and online communication and often use them to obtain information and guidance about mental health concerns [28]. The internet also offers a platform for young adults to exercise autonomy and seek support for complex and sensitive issues [29].

Factors that influence adults’ willingness to engage with electronic services include prior experience with online platforms, confidence in using technology, perceived need for assistance, and the effectiveness and user-friendliness of apps [30]. Researchers have also examined students’ perspectives on digital mental health interventions (DMHIs) in college settings, and findings suggest promising directions for implementation [31].

## Technology-Based Interventions: Emergent Directions

A variety of technology-based interventions are being used and studied in the field of mental health. Examples include teletherapy; computerized, internet-based interventions; self-help mobile apps (including gamified tools); artificial intelligence (AI)–powered chatbots; virtual reality therapy; and online supportive communities. Solutions such as teletherapy have also been examined and there is some evidence to suggest that teletherapy achieves effectiveness comparable with in-person therapy for several conditions, including depression and anxiety [32].

Evidence supporting web-based and mobile app–based psychological interventions continues to grow for concerns such as stress, distress, depression and anxiety, eating disorder symptoms, and enhancement of well-being among students [33,34]. These programs have evaluated multiple approaches, including cognitive behavioral therapy (CBT), acceptance and commitment therapy, mindfulness-based techniques, and skills training delivered through digital platforms.

A systematic search of reviews and meta-analyses on DMHIs for university students found that web-based (computer-delivered) interventions were effective or at least partially effective in reducing depression, anxiety, stress, and eating disorder symptoms; similar findings were reported for mobile apps [35]. Another systematic review and meta-analysis of studies on the effectiveness of digital interventions for university students with ongoing mental health difficulties showed medium effect sizes for both depression and anxiety [36].

In addition to treatment, researchers have examined preventive and promotive interventions [37]. One review analyzed 52 publications from 15 countries on 48 mobile apps designed to promote positive mental health. It identified 39 randomized controlled trials and found some evidence supporting the effectiveness of individual apps for fostering well-being [38].

Technology-based platforms can also serve as a means to improve mental health literacy and reduce stigma. For example, while excessive social media use is associated with adverse mental health outcomes, studies also highlight its potential to promote mental well-being by raising awareness, building supportive networks, and offering early access to help for individuals in distress [39,40]. A qualitative study of youth described 3 features of social media consumption—social connection, engagement with content, and opportunities for self-expression—that may contribute to positive mental health [39]. A review of media-driven mental health awareness campaigns for individuals aged 10 to 24 years identified evaluations of 15 initiatives across 8 countries and reported generally positive changes in attitudes, beliefs, intentions (eg, reduced stigma), and behaviors (eg, increased help-seeking) [40].

Online anonymous screening programs are as yet another use of technology to reduce negative attitudes and stigma-related concerns among young adults [41]. Available research suggests that distressed young adults may prefer online modes for initial contact, which can increase subsequent willingness to pursue in-person services after initial online engagement [42]. Smartphones have also supported mental health education and peer support programs aimed at empowering communities and cultivating well-being [42].

There is also emerging literature on technology-enabled strategies to increase help-seeking among distressed individuals [43]. Targeted strategies to encourage help-seeking for mental health challenges appear effective for adults who are at risk or already experiencing difficulties [44].

Most research on DMHIs has originated in high-income settings. A review of 49 studies assessed mobile, online, and other remote technologies for treatment and prevention of mental disorders in LMICs and found that most studies focused on feasibility and acceptability and the results suggested that these hold promise for effectiveness. These studies encompassed various apps such as tools to enhance clinical care and train health workers, mobile solutions to identify mental disorders, digital platforms to improve treatment adherence and recovery, online self-help programs for individuals with mental health conditions, and programs for substance use prevention and management [45].

In India, research on technology for mental health has increased over the last decade. With the advent of the COVID-19 pandemic, state and central government initiatives expanded efforts to address mental health needs [46], and the field of telemedicine also advanced rapidly [47]. Studies from India show a gradual shift from screening-based electronic decision support systems, task-sharing models to increase contact coverage, and text message–based interventions to web-based and mobile app–based programs and integrated delivery modalities [48-55]. A WhatsApp-based intervention has shown effectiveness in reducing barriers and improving help-seeking inclination and behavior among distressed young adults, demonstrating promise as a scalable approach [56]. Technology-enabled training for the mental health workforce has also grown substantially [57].

The Government of India’s Tele-Mental Health Program, Tele-MANAS (Tele Mental Health Assistance and Networking Across States) represents a national milestone in using technology to reduce the treatment burden and expand access to care [58]. However, additional research is needed to understand attitudes, preferences, and acceptability of DMHIs in low-income countries and among marginalized and underserved young people [59,60].

## Technology for Mental Health in Higher Education Settings

Multiple barriers such as a preference for self-reliance, low perceived need, limited awareness of available services, understaffed on-campus student well-being services, scheduling constraints and conflicts, waitlists due to limited appointment availability, time constraints, low confidence in service utility, stigma, discomfort with professional visits, and confidentiality concerns can impede help-seeking even when services are available on campuses [61-63]. These observations have provided an impetus to the use of technology-based mental health interventions in universities worldwide.

Technology-based tools may include websites, mobile apps, and online platforms and forums to name a few. Some are unguided self-help resources (fully self-directed tools with no human support); while others incorporate human support that can range from minimal guidance by trained laypersons (via text, phone, or email) to blended formats that combine digital self-help with face-to-face or online clinician-delivered support (eg, therapy sessions). Unguided or minimally guided interventions may be helpful for mental health promotion and milder problems with less acute needs; self-guided DMHIs typically produce small effects [64,65]. More human resource–intensive, blended interventions may be appropriate for individuals with more severe symptoms or complex problems [55].

Young people frequently use the internet to seek information that helps them understand and address personal and health concerns [17,28]. This pattern has mixed implications. Despite frequent online searches about mental health, many students remain unaware of internet-based interventions [66]. In one study, students who searched online showed enhanced mental health literacy—greater optimism about psychotherapy and reduced stigma in some domains—but lower willingness to seek or recommend professional help [67]. A review of university students’ perceptions and experiences with telemental health reported that students generally view it as convenient, accessible, user-friendly, and beneficial, with potential to mitigate stigma-related barriers [68]. Preferences for internet-based interventions with higher levels of human interaction, video content featuring humans have also been described [66].

Educational institutions can deploy technology-based platforms across the spectrum of prevention and care: universal interventions for all students, selective interventions for those at risk, and indicated interventions for students with significant symptoms. These efforts can support prevention, mental health promotion, treatment, and crisis response. Indicated interventions show the largest effects, particularly when participants have access to human support during the intervention, either in-person or via digital channels such as email or online contact [69].

A review of digital interventions for depression, anxiety, and psychological well-being among college students assessed effectiveness, usability, acceptability, uptake, and adoption [34]. Of 89 included studies, most interventions were website-delivered, with internet-based CBT being the most common approach. Many programs incorporated human support in the form of coaching; most demonstrated full or partial effectiveness on psychological outcomes, although nearly half did not report usability or acceptability. A separate systematic review evaluated the reach (percentage of invited students who expressed interest) and uptake (percentage of enrolled students who initiated an intervention) of CBT-based DMHIs for college students [21]. Of those studies that reported reach or uptake, most had evaluated unguided or guided self-help programs. Although the overall reach of DMHIs was found to be low as operationalized, enrolled participants demonstrated high uptake. Despite evidence that improving reach and uptake could increase public health impact, most studies did not report either outcome [21].

Another systematic review of 27 randomized controlled trials of technology-based interventions addressing depression, anxiety, and related concerns (excluding substance misuse and eating disorders) found that approximately half of the interventions yielded at least one significant positive outcome versus controls at postintervention. The review emphasized both the promise of such interventions in university settings and the need for more high-quality trials [70]. Digital technologies may also contribute to suicide prevention, although they are unlikely to be sufficient as stand-alone interventions and appear most effective when they complement ongoing clinical care [71].

It has been observed that DMHIs commonly used on campuses often lack robust, student-specific effectiveness data, underscoring the need for evaluations across diverse student populations and institutional contexts [72,73]. Additional research is needed to examine drivers and enablers of user uptake and engagement.

A comprehensive review examined 9 specific DMHIs offerings identified from a random sample of 200 colleges and universities in the United States through examining institutional website analyses and conducting interviews with administrators and experts from 20 institutions [73]. Interventions were grouped as multicomponent programs, self-guided programs, and connector programs. It was observed that states or colleges often purchase access for students to use these tools at no cost. Also, this review noted that some interventions were not designed specifically for college students but were widely used by them and frequently recommended by institutions. DMHIs have been implemented for screening, improving mental health literacy, and encouraging at-risk students to seek appropriate support. Other examples include online support groups, message boards, module-based web interventions, and skills-building apps focused on resilience, coping, and mindfulness.

Despite growing availability, a research-to-practice gap persists: technology-enabled interventions remain underused on many campuses, and few studies have evaluated their implementation within college counseling or wellness centers [74-77]. A systematic review of university students’ digital use for mental health found that, although students are ready to use digital platforms for mental health and well-being, they face challenges related to discerning the trustworthiness of information and data safety [78]. In addition, the potential cost-effectiveness of DMHIs in college and university settings remains an area that is yet to be examined systematically [73].

## Indian Education System: Challenges and Opportunities in the Use of Technology for Mental Health

India has one of the largest education systems in the world, with 1168 universities and university-level institutes, 45,473 colleges, and 12,002 stand-alone institutes, including 165 institutes of national importance. On average, there are 30 colleges per lakh (100,000) eligible persons aged 18-23 years in India [79]. In 2021-2022, 78% of colleges in India were found to be privately operated and 66% of total college enrollment occurred in these institutions, indicating higher education costs for students [80]. Total enrollment in higher education was nearly 43.3 million in 2021-2022, according to the All India Survey on Higher Education [79]. The National Education Policy (NEP) aims to increase the gross enrollment ratio to 50% by 2035, which would place a substantially larger proportion of the eligible population in the higher education system and has implications for ensuring accessible mental health support across institutions [81].

India has experienced rapid digitalization across many domains, including higher education (eg, course orientations, electronic learning, content sharing, evaluation and feedback systems, and online guidance). As one of the world’s most populous nations with a substantial youth population, India faces unique opportunities and challenges in education and health, including providing quality higher education and health access to a large and diverse student body. The higher education sector is increasingly integrating technology to support remote learning and digital classrooms. The expansion of internet connectivity and mobile technology has accelerated the rise of online learning platforms, virtual classrooms, and Massive Open Online Courses, allowing students to access materials and participate in interactive learning [82,83].

Despite notable progress in strengthening higher education in India, several challenges exist that have a bearing on student mental health and well-being. Two interrelated issues are employment opportunities and an exam-centric system. According to the International Labour Organization, more than half of employed graduates in India work in low-skilled jobs, and fewer than one-third hold positions requiring high-level skills; many highly educated young adults, including those with technical training, are overqualified for their jobs [84].

India’s examination-centric education system has been widely criticized for prioritizing rote learning over competency and skill development, influencing career paths and contributing substantially to student stress. The National Eligibility cum Entrance Test and the Joint Entrance Examination are among the most competitive entrance examinations, viewed as critical steps toward admission to medical and engineering programs. Millions of students compete for a limited number of seats in top institutions, fueling intense pressure and stress. This dynamic has also driven the growth of a coaching industry focused on examination preparation, with students sometimes investing years in study and attending coaching institutes for extended periods [85].

These pressures carry financial, social, and health consequences. Rigorous preparation schedules and frequent mock examinations often reduce time for social interaction, physical activity, and self-care, while increasing risk for anxiety and depressive symptoms because of self-doubt, overwhelming stress, fear of failure, setbacks, uncertainty about career progress, guilt related to perceived underperformance, concerns about disappointing or burdening families, and a low sense of support [85,86]. These trends—combined with employability challenges, societal norms that prize a narrow range of careers, the high prevalence of common mental health concerns (including subthreshold symptoms) among young adults, a shortage of mental health professionals, and low awareness and stigma around help-seeking—further exacerbate the mental health situation in Indian higher education.

## Potential for Using Technology to Support Mental Health in Indian Institutes of Higher Education and Key Considerations: Policy Support and Opportunities for Integration

The University Grants Commission of India has issued Guidelines for the Promotion of Physical Fitness, Sports, Students’ Health, Welfare, and Psychological and Emotional Well-being [87]. These guidelines recommend that every institute of higher education establish a Student Services Centre to provide counseling, guidance, and mental health services in both online and offline formats, including telehelp [87].

India’s NEP aims to enhance student learning outcomes, improve teacher training, and strengthen educational infrastructure [81]. From 2013-2014 to 2020-2021, combined state and central government education expenditure ranged from 3.9% to 4.6% of gross domestic product; the NEP recommends raising education spending to at least 6% of gross domestic product [88]. Several NEP recommendations could function as protective factors for student well-being, including a shift toward continuous assessment rather than exclusive emphasis on end-of-course examinations and multiple entry and exit options in undergraduate programs that allow students to realign academic trajectories based on interest and opportunity [88]. The NEP’s emphasis on digital tools and online learning platforms creates opportunities to not only enhance educational access and academic performance but also to expand access to mental health support through technology-enabled interventions.

The NEP’s governance vision—independent institutional boards composed of highly qualified members, greater academic and administrative autonomy, and a “light but tight” regulatory framework under a single higher education regulator—may help embed mental health initiatives into institutional offerings. However, there are likely to be implementation challenges including resource constraints, acceptability and receptivity among stakeholders, and administrative bottlenecks.

India’s National Suicide Prevention Strategy, released in 2022, is another policy document of high relevance. It recognizes suicide as a major public health concern and sets a goal of reducing suicide mortality by 10% by 2030 [89]. The strategy calls for a multisectoral approach and highlights the need for multipronged approaches including efforts to raise awareness of common mental health concerns, reduce stigma, strengthen psychosocial support, train key stakeholders (eg, gatekeeper training of teachers) for early risk identification and referral, expand the mental health workforce, improve help-seeking among persons in distress, and bolster supports for individuals experiencing suicidal crises and their families and using youth engagement programs to name a few. [89]. Many of these recommended activities could benefit from technology-based platforms for scalable implementation.

The recent Digital Personal Data Protection (DPDP) Act has direct implications for mental health data privacy in India [90]. It seeks to protect personally identifiable mental health information from misuse, discrimination, and exploitation and mandates that the data fiduciaries (the entities processing the data) must obtain informed consent from individuals and should process data for specified purposes and adhere to principles such as data minimization and retention. Moreover, individuals have the right to withdraw consent, which obligates data fiduciaries to stop collecting, storing, using, or sharing data unless permitted under the Act or other relevant applicable laws [90]. Complexities in operationalizing these provisions (particularly within [Institutes of Higher Education] IHEs) require careful consideration. In parallel, the Indian Council of Medical Research has issued Ethical Guidelines for Application of AI in Biomedical Research and Health Care, which outline principles for ethical development, deployment, and governance of AI-based solutions in research and clinical practice, emphasizing accountability, autonomy, data privacy, risk minimization, and fairness [91]. While there is no comprehensive regulatory body covering mental health apps, a number of legal and regulatory frameworks would be relevant depending on the nature of the digital intervention. Going forward, it would be useful to evolve a uniform policy on the implementation of technology-based platforms for mental health in educational settings based on applicable legal and regulatory frameworks.

## Implementation Challenges and Enabling Measures for DMHIs in Higher Education

Both digital and in-person interventions for college students may pose somewhat distinct barriers to their usage. While barriers such as procedural hurdles in scheduling, including time constraints, financing, lack of trust in confidentiality, fear of stigma or social consequences, become salient for in-person interventions, content-related dissatisfaction, unmet needs for direct human communication, and privacy concerns are some of the key deterrents to digital interventions experienced by students [92].

A review of research from 2019 to 2024 on DMHIs for college students revealed that despite promising psychological outcomes and good adherence, there was only modest uptake, with most studies originating from the United States or Canada and featuring predominantly female samples [93]. A scoping review of DMHIs for adolescents in LMICs found that while fear of exposure, stigma, and low awareness remained significant barriers, factors such as free access, confidentiality assurances, culturally appropriate content, and inclusion of human-led options facilitated DMHI engagement [94]. A Delphi study to attain expert consensus on barriers and strategies to improve uptake of DMHI for college students from diverse and marginalized backgrounds in the United States indicated strategies such as co-design processes, user-customizable privacy settings, content personalization, simplified onboarding, and embedding DMHIs within campus ecosystems. The study also highlighted the value of emphasizing autonomy, flexibility, and inclusion of representatives from the target groups to enhance acceptability and uptake of digital interventions [95].

There is a notable dearth of research in LMICs on the development and implementation of digital interventions, with an even greater gap in the context of IHEs. While there remains an urgent need for hybrid effectiveness–implementation studies to address the significant research gap in LMICs [93], existing international evidence on barriers and facilitators can help guide context-sensitive strategies for Indian higher education settings. The acceptability and uptake of digital interventions, as well as engagement with them, may be enhanced by measures such as student involvement across development, deployment and evaluation phases, transparent information on the scope of digital interventions and privacy policies, and customizable privacy controls that signal safety and credibility [95]. Offline-first apps which can perform core functions without internet access, may help minimize barriers such as bandwidth limitations and intermittent connectivity [96]. Emphasizing autonomy, flexibility, and representation of target groups in campaigns that build awareness about digital and in-person interventions may further increase their appeal among diverse groups, including marginalized students and reduce barriers to help-seeking [95]. A simplified onboarding process, clear in-app explanations of features and risks, and human support (such as brief coaching or scheduled check-ins) can mitigate barriers related to digital literacy for college students. Scope for content personalization, adoption of stepped-care models that offer varying levels of intervention intensity based on individual needs, and integration of DMHIs within campus ecosystems—such as counselling centers, and peer support networks can enhance student engagement with mental health services [73,97]. These efforts are more likely to succeed when paired with clear, accessible messaging about available support and referral pathways, helping students navigate both digital and in-person options with greater ease [20,93].

Linguistic and sociocultural diversity is another challenge in implementing DMHIs in Indian IHEs. Even when mental health content is translated, complex terminology can impede understanding. A few practical language-access strategies can improve comprehension and engagement. Approaches such as using locally meaningful terms, culturally appropriate adaptations, and offering human support to help students navigate app content can be helpful. Given the prominence of English in higher education alongside regional languages, and frequent code-switching in everyday student communication, mixed-language content tailored to context may be more effective than strictly monolingual formats in DMHIs. Incorporating audio content in local languages, delivered in a conversational style, may further enhance accessibility, and this warrants further research.

The rapid proliferation of mental health apps, often developed with minimal involvement from qualified mental health professionals [98], raises concerns about the choice of digital tools to be deployed in IHEs, keeping in view their validity, safety, and user trust. Ethical issues related to potential harm, inadequate safeguards, and unclear accountability can further complicate implementation [99-101]. Additionally, the financial burden of sustaining these interventions at scale, especially in resource-constrained settings, presents a significant barrier. These issues are discussed in greater detail in the subsequent section on strategic government-level action.

## Reflections on Strategic Actions Across Levels: Key Considerations for IHEs

### Data Governance and Terms of Use

IHEs should establish clear data governance policies and accountability mechanisms to ensure a safe digital environment for students seeking digital mental health services. Students must receive transparent, easy-to-understand information about ownership and use of personally identifying information and sensitive mental health data. Institutions should implement specific, clearly articulated terms and conditions and consent protocols [20]. These should address, at a minimum, conditions for any data sharing with the institution; the scope and limits of the intervention; and the use of AI and its limitations. To enhance safety on online mental health platforms, institutions should adopt appropriate data-handling policies, establish risk-management protocols for monitoring signs of distress, and proactively offer support when needed.

### Positioning of DMHIs and Integration with In-Person Services

Current evidence on DMHIs in IHEs, notable gaps in mental health and digital literacy as well as expert consensus, suggests that these interventions are best positioned as additions or supplements to face-to-face professional services rather than as stand-alone substitutes [73,102-104]. Framing DMHIs in this complementary role can also enable the development of stepped-care models, where students can access interventions of increasing intensity based on their needs and blended approaches that combine digital tools with in-person professional support, both of which are emerging as promising strategies within IHEs [20,55]. Accordingly, digital offerings should incorporate features such as timely, algorithm-driven nudges to encourage professional care when indicated [98]; clear crisis-support guidance and multiple help-seeking options; integration with offline services; and transparent communication about the platform’s capabilities and limitations.

### Ensuring Content Quality of DMHIs

The rapid proliferation of mental health platforms and mobile apps—varying widely in quality, purpose, and clinical grounding—can overwhelm and confuse users, while rigorously evaluated tools often remain out of reach [60,98]. Until stronger evidence accumulates, IHEs could prioritize platforms that are developed or vetted by qualified mental health professionals and include clinical oversight to the extent feasible. This approach can help ensure delivery of evidence-informed content, minimize potential harm, and maximize therapeutic benefit for the users.

### Collaborative Engagement With Students Across Program Phases

Active student involvement in the development, decision-making, and implementation of mental health programs and services—both digital and offline is essential for their success [17,20,73]. Service offerings perceived as tokenistic, externally imposed, or disconnected from an ethos of shared responsibility for student well-being are unlikely to be adopted. To support meaningful collaboration, IHEs should engage students across all phases—from co-design to rollout and evaluation and incorporate regular feedback mechanisms, such as periodic surveys, to refine services and digital tools, from users as well as nonusers of services.

### Provisioning for a Range of Apps in IHEs

Institutions should plan for training platforms and resources for both educators and students, as outlined in the sections below.

#### Use of Online Platforms for Regular Orientation and Training for Leaders and Faculty

Leadership development, facilitation, and sensitization—together with training that equips faculty to support socioculturally diverse student groups—are important components of student mental health programs [105,106]. Faculty training for student mental health and suicide prevention may include mentoring vulnerable students; recognizing suicide risk and warning signs; and establishing processes for early identification, first-line support, crisis intervention, and appropriate referral. Although technology platforms in Indian higher education currently focus on teaching, learning, and evaluation—as well as on planning and administrative processes—these platforms can be adapted to deliver structured modules that help educators and leaders understand student mental health needs, provide tools for support, and guide them in mentoring, risk assessment, and offering assistance for students in distress.

#### Spectrum of Technology-Based Resources for Student Mental Health

IHEs can deploy a diverse range of technology-based mental health initiatives that offer students a sense of choice and autonomy and allow them to engage with supports aligned to their felt needs, preferences, and readiness for help. These may include (1) centralized digital repositories of mental health resources in multiple formats and languages, regularly updated and publicized. (2) Interactive platforms to build mental health literacy, reduce stigma, and foster inclusion and belonging. (3) Anonymized self-screening tools with tailored recommendations on intervention intensity and access pathways. (4) Peer support training tools and moderated online forums to strengthen student-led support systems. (5) Low-intensity chat-based support, including AI-assisted options, moderated by trained nonspecialists. (6) Minimally guided self-help tools for common concerns and personal well-being goals. (7) Targeted digital outreach (eg, via WhatsApp) to reduce help-seeking ambivalence among distressed, non–treatment-seeking students). (8) Blended care models combining digital self-help with online or offline therapist sessions for students needing higher-intensity support. (9) Access to telehelplines offering crisis support and brief interventions.

Beyond access to different kinds and intensities of interventions, offering format choices (eg, text- vs audio-based content) and options for communicating with a trained human agent (eg, chat- vs phone-based support) can increase the appeal of DMHIs for college students. The approaches listed above need to be integrated within campus ecosystems and aligned with broader mental health strategies rather than operate in isolation. Whenever feasible, these could be loosely integrated or “bundled” to allow students to move from one to another and exercise rights to indicate preferences and consent for transferring personal data between apps to improve efficiency. Students should be empowered to exercise their rights by explicitly indicating preferences and providing informed consent for any transfer of personal data between apps or services. This can facilitate both efficiency and respect for autonomy, including the right to opt in or out of such data-sharing arrangements. In addition to user-initiated transitions across levels, DMHIs need to incorporate algorithmic nudges that prompt students to consider interventions that are more in alignment with the nature and severity of their concerns. This function of DMHIs becomes critical in the context of low mental health literacy.

## Indicators of Success

The implementation knowledge base benefits from systematic use of metrics that capture delivery realities alongside clinical outcomes and provide leads for further planning. Campuses can track practical indicators when embedding DMHIs within institutional support systems. Reach, adoption, effectiveness, implementation, and maintenance (often referred to collectively as RE-AIM) are widely used parameters for evaluating DMHIs for college students [93]. When interventions are integrated into campus mental health services, indicators of success may include increased overall uptake of services; the proportion of previously distressed, non–treatment-seeking students who initiate low-intensity digital supports (particularly those designed to enhance help-seeking); sustained engagement over time; user satisfaction; feasibility and acceptability among stakeholders; and evidence of sustainability within the campus context, in addition to relevant effectiveness outcomes.

## Key Considerations for Government Action

1. Developing a coherent set of national guidelines for mental health apps—both broadly and for use within higher education can incentivize local developers to work toward creating quality solutions. For IHEs, such guidelines should specify minimum standards for content quality, mechanisms for independent quality checks, and safeguards for user safety. Clear protocols for data governance, privacy, and security and regulatory oversight to ensure ethical deployment in IHEs would also be essential.
2. In a country like India, with a vast population of college-going young adults and a rapidly growing number of newer digital apps and services entering the marketplace, it can become very confusing and overwhelming for inclined users to find an app that would suit their needs [98]. A centrally managed navigator platform that curates evaluated options, publishes plain-language summaries, and helps students compare and select appropriate digital interventions can ease the burden on the users to find what may suit their needs. Beyond navigation, such a comprehensive, youth-focused digital platform across IHEs could also serve as an essential resource to provide credible self-help materials in multiple languages, and feature a directory of affordable mental health services for students. Additionally, such a platform could include positive peer experiences of help-seeking and thus play a role in shaping peer norms around help-seeking for mental health.
3. Scarcity of trained mental health professionals in India, coupled with observations on low uptake of counselling services in IHEs and students’ preference for online mediums, highlights the utility of blended offerings that combine offline services with digital tools. Training of mental health service providers in IHEs in the effective and ethical deployment and integration of DMHIs should be prioritized as part of a coordinated government-led capacity-building initiative. This effort can itself leverage online training modules to ensure scalability and reach.
4. Recognizing and incentivizing IHEs through accreditation, or awards based on well-functioning student mental health support systems, can encourage greater investment in mental health initiatives including DMHIs, and lead to the development of best practices and improved adoption.
5. To operationalize the above-mentioned directions at a national scale, structured multistakeholder collaborations are warranted. Lack of coordinated involvement of key actors (eg, academics, health providers, end users, and private industry) has been identified as a barrier to implementing digital mental health solutions [107,108]. India’s digital mental health landscape similarly reflects fragmented growth, with isolated efforts from academia, government, private industry, and individual innovators [109]. Consolidating these efforts through formal collaboration can address fragmentation and accelerate the responsible scale-up of DMHIs for IHEs in India.
6. Reviews of DMHIs in IHEs have highlighted a consistent gap in reporting on implementation adaptations and cost-related data—even in studies conducted in high-income settings [73]. DMHIs vary in cost and cost-effectiveness depending on their nature and context of implementation [109,110]. Moving forward, it is critical to examine how DMHIs can be strategically integrated with in-person services to optimize cost-efficiency and enhance the overall effectiveness of mental health support systems. Mandates by the government to report costs, uptake and engagement, and satisfaction with mental health services by IHEs can improve this scenario for the government to evolve strategies for optimizing resources while expanding access to effective mental health support for students.
7. Sustainable financing is critical to expanding access to student support systems within IHEs. This could include increased public investment in mental health, establishment of dedicated budget lines for student-mental health services, and the exploration of blended-finance models that combine government, philanthropic, and institutional resources to ensure long-term viability and reach. Public-Private-Partnership (PPP) models are recognized facilitators for integrating digital technologies within mental health systems [111]. In India, relevant precedents include PPPs in the National Dialysis Programme, Gujarat’s Chiranjeevi Yojana, and telemedicine initiatives such as Apollo Telehealth in collaboration with state governments [112-114]. Applied to DMHIs for higher education, PPPs can balance public interest with commercial viability, enable evidence-informed planning, and support sustainable implementation costs through shared resourcing. This would require a national liaison or coordination agency (or an existing national body assigned this role) which could serve as a single point of contact among stakeholders.
8. Amid rising concerns around student mental health in India, underscored by the recent Supreme Court of India directives for IHEs regarding access to mental health services [115], there is an urgent need for dedicated funding to support national-level implementation–effectiveness research on integrated mental health strategies, including DMHIs. This effort should be embedded within a long-term operational framework, rather than a time-bound project, to ensure sustained progress in research, policy development, and system-wide integration.

On the whole, a multitiered framework of technology-based, evidence-informed mental health resources, codeveloped with students, tailored to sociocultural contexts, and integrated with mainstream in-person services at key touchpoints can significantly strengthen support systems and address the diverse needs of students in IHEs. To ensure sustained impact, this approach must be supported by coordinated national leadership, collaboratively evolved guidelines, shared financing mechanisms, including public-private partnerships, and long-term implementation–effectiveness research to address persistent gaps in student mental health care across India’s higher education landscape.

## Abbreviations

AI: artificial intelligence
CBT: cognitive behavioral therapy
DMHI: Digital Mental Health Intervention
DPDP: Digital Personal Data Protection
DSM-IV: Diagnostic and Statistical Manual of Mental Disorders (Fourth Edition)
IHEs: Institutes of Higher Education
LMIC: low- and middle-income country
NEP: National Education Policy
PPP: Public-Private-Partnership
WHO: World Health Organization
